# Supplementary figures and images for: Six-month post-intensive care outcomes during high and low bed occupancy due to the COVID-19 pandemic: A multicenter prospective cohort study
Source: PLoS One. 2023 Nov 16;18(11):e0294631. doi: 10.1371/journal.pone.0294631 (PMC10653414; doi:10.1371/journal.pone.0294631)

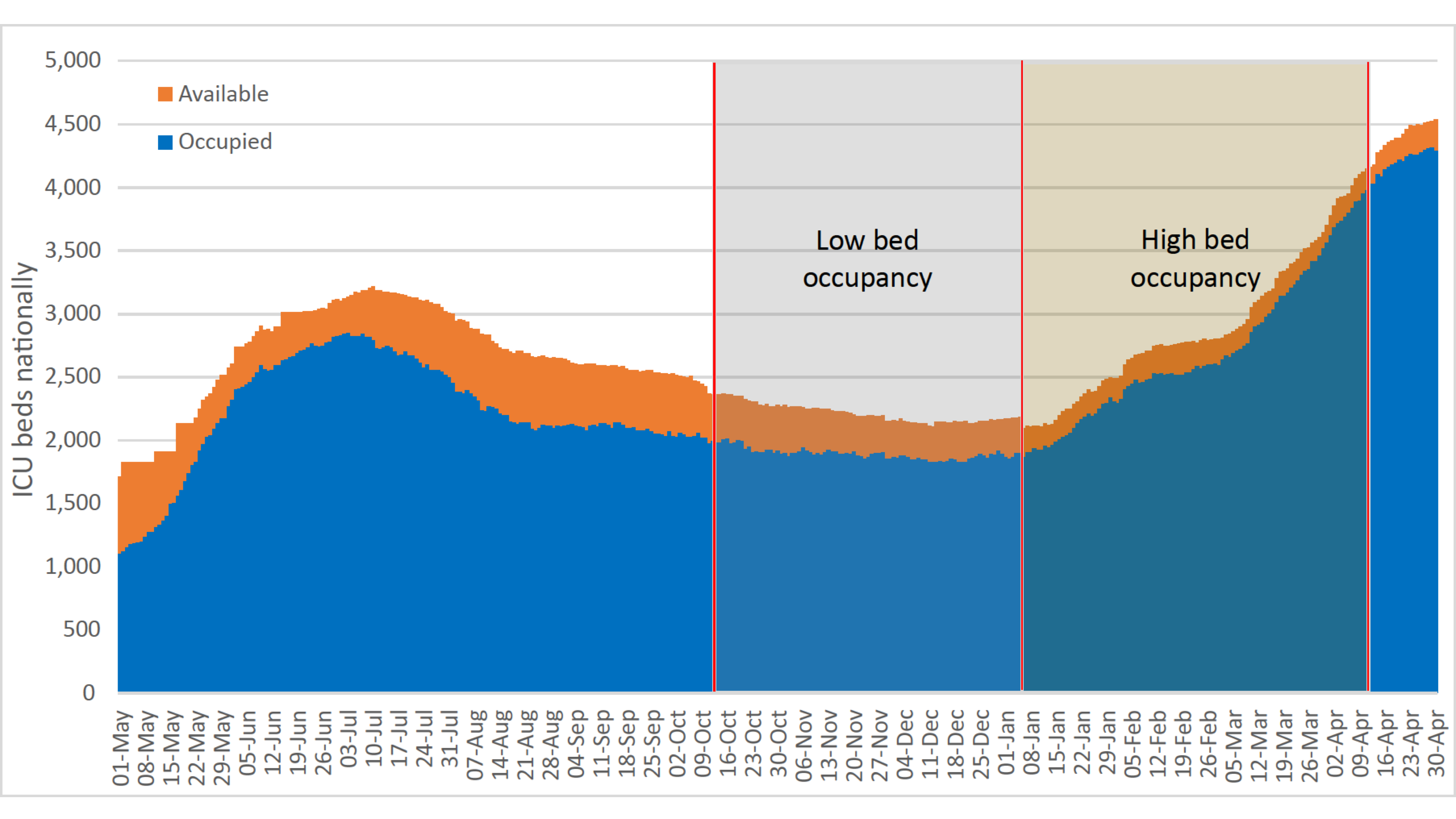

Supplement: S1 Fig — Data retrieved from www.minciencia.gob.cl/COVID-19 and plotted by the authors. (TIFF) [file pone.0294631.s001.tiff]
