# Supplementary material for: Six-month post-intensive care outcomes during high and low bed occupancy due to the COVID-19 pandemic: A multicenter prospective cohort study
Source: PLoS One. 2023 Nov 16;18(11):e0294631. doi: 10.1371/journal.pone.0294631 (PMC10653414; doi:10.1371/journal.pone.0294631)
Supplement: S1 Table — (DOCX) [file pone.0294631.s002.docx]

**S1 Table. Description of the measurement instruments used in the IMPACCT COVID-19 Study**

| **Outcome domain** | **Measurement instrument** | **ICF domain** | **Scoring range** | **Categories/Interpretation** | **Evaluation time point** |
| --- | --- | --- | --- | --- | --- |
| Disability | WHO Disability Assessment Schedule (WHODAS 2.0) | Activities and Participation (d) | Total score 0–48; converted to a percentage 0–100% (Standardized disability level) | No disability (<5%) Mild disability (5–24%) Moderate disability (25–49%) Severe disability (50–95%) Complete disability (>95%) | ICU discharge, 3- and 6-months post ICU |
| Frailty (prior to ICU admission) | Clinical Frailty Scale (CFS) | Activities and Participation (d) | Very fit (1) Well (2) Managing well (3) Vulnerable (4) Mildly frail (5) Moderately frail (6) Severely frail (7) Terminally ill (8) Very Severely Frail (9) | Frail (>4) | ICU discharge |
| Cognitive function | Montreal Cognitive Assessment-blind (MoCA–Blind) | Global cognition (b164) | 0–22 points | Cognitive impairment (<18) | ICU discharge, 3- and 6-months post ICU |
| Depression | Hospital Anxiety and Depression Scale (HADS–D) | Emotional functions (b152) | 0–21 points | Normal (0–7) Borderline abnormal (8–10) Abnormal (>11) | ICU discharge, 3- and 6-months post ICU |
| Anxiety | Hospital Anxiety and Depression Scale (HADS–A) | Emotional functions (b152) | 0–21 points | Normal (0–7) Borderline abnormal (8–10) Abnormal (>11) | ICU discharge, 3 and 6-months post ICU |
| Post-traumatic stress | Impact of Event Scale-Revised (IES-R) | Emotional functions (b152) | 0–88 points | Normal (0–23) Some PTSD symptoms (24–32) Likely diagnosis of PTSD (33–36) PTSD (>36) | ICU discharge, 3- and 6-months post ICU |
| At least one mental, physical or cognitive impairment | WHODAS 2.0, MoCA-Blind, HADS-D, HADS-A, IES-R | Functions, Activities and Participation (d) | n/a | Any of the following:  Severe or complete disability (>49%)  Cognitive impairment (<18)  Clinically significant depression (>7)  Clinically significant anxiety (>7)  PTSD symptoms (>32) | ICU discharge, 3- and 6-months post ICU |
| Health-related quality of life | European Quality of Life Health Questionnaire 5 Domains (EQ-5D-3L) | Activities and Participation (d) | -0.8 to +1 (Chilean utility score) | 1 = full health; and 0 = a state as bad as being dead. Values <0 indicate health states worse than a state similar to being dead | 3- and 6-months post ICU |
| Peripheral muscle strength | Medical Research Council Sum Score (MRC-SS) | Muscle function (b7300) | 0–60 points | Without ICU-AW (≥48) Significant ICU-AW (36–47) Severe ICU-AW (<36) | ICU discharge |
| Mobility | Functional Status Score for the Intensive Care Unit (FSS-ICU) | Mobility (d4) | 0–35 points | Higher scores indicate better mobility | ICU discharge |
| Education level | Years of formal education completed | Education (d810-d839) | n/a | <9 years 9 to 12 years >12 years | ICU discharge |
| Employment | Employment status | Work and employment (d840-d859) | n/a | Employed–Full Time  Employed–Part Time  Unemployed Retired | ICU discharge, 3- and 6-months post ICU |

Definition of abbreviations: ICF = International Classification of Functioning, Disability and Health; WHO = World Health Organization; ICU = intensive care unit; PTSD = Post-Traumatic Stress Disorder; ICU-AW = intensive care unit acquired weakness.
