# Supplementary material for: Six-month post-intensive care outcomes during high and low bed occupancy due to the COVID-19 pandemic: A multicenter prospective cohort study
Source: PLoS One. 2023 Nov 16;18(11):e0294631. doi: 10.1371/journal.pone.0294631 (PMC10653414; doi:10.1371/journal.pone.0294631)
Supplement: S2 Table — (DOCX) [file pone.0294631.s003.docx]

**S2 Table. Functional outcomes and employment status**

|  | **ICU discharge (*n=*252)** | **3-month follow-up (*n=*105)** | **6-month follow-up (*n=*67)** | ***p-*value** |
| --- | --- | --- | --- | --- |
| WHODAS–Standardized disability level, % | 27.8 (10.4–48.3) | 10.9 (4.5–26.8) | 9.8 (2.1–23.2) | <0.001 |
| WHODAS–Total score | 73 (49–101) | 41 (30–53) | 37 (30–47) | <0.001 |
| Understanding & communicating | 20.8 (4.2–41.7) | 8.3 (0–25) | 12.5 (0–29.2) | 0.007 |
| Mobility | 25 (0–60) | 10 (0–40) | 5 (0–30) | <0.001 |
| Self-Care | 12.5 (0–50) | 0 (0–12.5) | 0 (0–0) | <0.001 |
| Getting along with people | 12.5 (0–35) | 5 (0–15) | 0 (0–10) | <0.001 |
| Life Activities: household | 12.5 (0–59.4) | 6.25 (0–25) | 0 (0–31.3) | <0.001 |
| Life Activities: work or school | 12.5 (0–71.9) | 18.8 (0–50) | 0 (0–28.1) | 0.110 |
| Participation in society | 37.5 (18.8–62.5) | 18.8 (15.6–37.5) | 12.5 (3.1–28.1) | <0.001 |
| WHODAS–Level of disability |  |  |  | <0.001 |
| No disability (<5%) | 37 (14.7%) | 33 (31.4%) | 27 (40.3%) |  |
| Mild disability (5–24%) | 78 (31.0%) | 40 (38.1%) | 27 (40.3%) |  |
| Moderate disability (25–49%) | 75 (29.8%) | 27 (25.7%) | 11 (16.4%) |  |
| Severe disability (50–95%) | 62 (24.6%) | 5 (4.8%) | 2 (3.0%) |  |
| MoCA–Blind | 16 (12–18) | 18.5 (16–21) | 21 (18–22) | <0.001 |
| Cognitive impairment (<18) | 181 (71.8%) | 38 (36.2%) | 11 (16.4%) | <0.001 |
| HADS–depression score | 5 (2–9) | 6 (5–10) | 7 (5–9) | <0.001 |
| Normal (0–7) | 178 (70.6%) | 60 (57.7%) | 41 (61.2%) | 0.15 |
| Borderline abnormal (8–10) | 37 (14.7%) | 23 (22.1%) | 15 (22.4%) |  |
| Abnormal (>11) | 37 (14.7%) | 21 (20.2%) | 11 (16.4%) |  |
| HADS–anxiety score | 8 (5–12) | 6 (4–9) | 6 (3–10) | <0.001 |
| Normal (0–7) | 107 (42.5%) | 65 (62.5%) | 45 (67.2%) | <0.001 |
| Borderline abnormal (8–10) | 49 (19.4%) | 19 (18.3%) | 9 (13.4%) |  |
| Abnormal (>11) | 96 (38.1%) | 20 (19.2%) | 13 (19.4%) |  |
| IES-R | 44 (26–56) | 20 (8–39.5) | 20 (6–43) | <0.001 |
| Normal (0–23) | 52 (20.6%) | 56 (53.8%) | 37 (55.2%) |  |
| Some PTSD symptoms (24–32) | 36 (14.3%) | 12 (11.5%) | 6 (9.0%) |  |
| Likely diagnosis of PTSD (33–36) | 20 (7.9%) | 9 (8.7%) | 5 (7.5%) |  |
| PTSD (>36) | 144 (57.1%) | 27 (26.0%) | 19 (28.4%) |  |
| EQ-5D-3L, utility score |  | 0.7 (0.56–0.8) | 0.78 (0.56–1) | 0.55 |
| Problems with mobility |  | 38 (37.3%) | 21 (32.3%) | 0.51 |
| Problems with personal care |  | 14 (13.5%) | 9 (14.1%) | 0.91 |
| Problems with usual activities |  | 37 (35.6%) | 23 (34.8%) | 0.92 |
| Problems with pain/discomfort |  | 60 (58.3%) | 38 (57.6%) | 0.93 |
| Problems with anxiety/depression |  | 49 (47.1%) | 29 (43.9%) | 0.69 |
| Current employment status |  |  |  | 0.26 |
| Employed–Full Time |  | 43 (41.0%) | 35 (53.8%) |  |
| Employed–Part Time |  | 16 (15.2%) | 5 (7.7%) |  |
| Unemployed |  | 31 (29.5%) | 14 (21.5%) |  |
| Retired |  | 14 (13.3%) | 11 (16.9%) |  |
| No answer |  | 1 (1.0%) | 0 (0.0%) |  |
| Change of employment |  | 71 (68.3%) | 40 (60.6%) | 0.39 |
| Type of employment change |  |  |  | 0.01 |
| Same job–fewer hours |  | 35 (49%) | 15 (38%) |  |
| Different job–same hours |  | 0 (0%) | 7 (18%) |  |
| Different job–fewer hours |  | 4 (6%) | 4 (10%) |  |
| Unemployed/Studying |  | 4 (6%) | 1 (3%) |  |
| Unemployed/ Stopped studying |  | 27 (38%) | 13 (33%) |  |
| No answer |  | 2 (3%) | 0 (0%) |  |

Definition of abbreviations: ICU=Intensive Care Unit; WHODAS = WHO Disability Assessment Schedule; MoCA-blind = Montreal Cognitive Assessment-blind; HADS = Hospital Anxiety and Depression Scale; IES-R = Impact of Event Scale-Revised; PTSD = Post-Traumatic Stress Disorder; EQ-5D-3L = European Quality of Life Health Questionnaire 5 Domains.

Data are median (quartile 1–quartile 3) or n (%). Percentages may not total 100 because of rounding.
