# Supplementary material for: Six-month post-intensive care outcomes during high and low bed occupancy due to the COVID-19 pandemic: A multicenter prospective cohort study
Source: PLoS One. 2023 Nov 16;18(11):e0294631. doi: 10.1371/journal.pone.0294631 (PMC10653414; doi:10.1371/journal.pone.0294631)
Supplement: S3 Table — (DOCX) [file pone.0294631.s004.docx]

**S3 Table. Baseline characteristics and intensive care unit outcomes at ICU discharge of patients assessed at 3 and 6 months**

|  | **Assessed at ICU discharge (*n=*252)** | **Assessed at 3 months (*n=*105)** | **Assessed at 6 months (*n=*67)** | ***p-v*alue** |
| --- | --- | --- | --- | --- |
| Age, years | 57 (46.5–67) | 57 (47–65) | 58 (49–65) | 0.90 |
| Female sex | 89 (35.3%) | 34 (32.4%) | 22 (32.8%) | 0.84 |
| Body mass index, kg/m2 | 29 (26–33.2) | 29.6 (27.4–33.4) | 29 (26–32.6) | 0.76 |
| Educational level |  |  |  | 0.97 |
| <9 years | 42 (16.7%) | 17 (16.2%) | 10 (14.9%) |  |
| 9 to 12 years | 68 (27.0%) | 28 (26.7%) | 16 (23.9%) |  |
| >12 years | 142 (56.3%) | 60 (57.1%) | 41 (61.2%) |  |
| Baseline employment status |  |  |  | 0.98 |
| Employed–Full Time | 147 (58.3%) | 65 (61.9%) | 39 (58.2%) |  |
| Employed–Part Time | 30 (11.9%) | 12 (11.4%) | 8 (11.9%) |  |
| Unemployed | 37 (14.7%) | 16 (15.2%) | 11 (16.4%) |  |
| Retired | 38 (15.1%) | 12 (11.4%) | 9 (13.4%) |  |
| Clinical Frailty Scale | 3 (2–3) | 3 (2–3) | 2 (2–3) | 0.66 |
| Very fit | 36 (14.3%) | 14 (13.3%) | 11 (16.4%) |  |
| Well | 84 (33.3%) | 37 (35.2%) | 26 (38.8%) |  |
| Managing well | 97 (38.5%) | 38 (36.2%) | 21 (31.3%) |  |
| Vulnerable | 22 (8.7%) | 11 (10.5%) | 5 (7.5%) |  |
| Mildly frail | 5 (2.0%) | 2 (1.9%) | 2 (3.0%) |  |
| Moderately frail | 7 (2.8%) | 3 (2.9%) | 2 (3.0%) |  |
| Severely frail | 1 (0.4%) | 0 (0.0%) | 0 (0.0%) |  |
| Charlson Comorbidity Index | 0 (0–1) | 0 (0–1) | 0 (0–1) | 0.92 |
| Admission diagnosis |  |  |  | 0.81 |
| Non–COVID-19 pneumonia or ARDS | 16 (6.3%) | 7 (6.7%) | 5 (7.5%) |  |
| COVID-19 pneumonia or ARDS | 191 (75.8%) | 83 (79.0%) | 52 (77.6%) |  |
| Abdominal surgery | 10 (4.0%) | 2 (1.9%) | 2 (3.0%) |  |
| Heart failure | 9 (3.6%) | 3 (2.9%) | 1 (1.5%) |  |
| Septic shock | 14 (5.6%) | 6 (5.7%) | 4 (6.0%) |  |
| Drug intoxication/suicide attempt | 3 (1.2%) | 0 (0.0%) | 0 (0.0%) |  |
| Cardiac arrest | 3 (1.2%) | 3 (2.9%) | 3 (4.5%) |  |
| Other | 6 (2.4%) | 1 (1.0%) | 0 (0.0%) |  |
| Organ System Supported during ICU stay |  |  |  |  |
| Advanced Respiratory Support | 252 (100.0%) | 105 (100.0%) | 67 (100.0%) | 1.00 |
| Basic Cardiovascular Support | 158 (62.7%) | 66 (62.9%) | 41 (61.2%) | 0.97 |
| Advanced Cardiovascular Support | 84 (33.3%) | 34 (32.4%) | 22 (32.8%) | 0.98 |
| Renal Support | 16 (6.3%) | 6 (5.7%) | 4 (6.0%) | 0.97 |
| Liver Support | 1 (0.4%) | 1 (1.0%) | 0 (0.0%) | 0.65 |
| Reintubated during ICU stay | 32 (12.7%) | 12 (11.4%) | 9 (13.4%) | 0.92 |
| Duration of ventilation, days | 9 (6–15.5) | 10 (6–14) | 10 (6–14) | 0.95 |
| ICU length of stay, days | 15 (10–25) | 14 (10–24) | 15 (10–24) | 0.99 |
| MRC-SS at ICU discharge | 52 (45–57) | 51 (44–58) | 52 (44–60) | 0.64 |
| Without ICU-AW (MRC-SS ≥48) | 167 (66.3%) | 71 (67.6%) | 48 (71.6%) | 0.90 |
| Significant ICU-AW (MRC-SS 36–47) | 71 (28.2%) | 29 (27.6%) | 17 (25.4%) |  |
| Severe ICU-AW (MRC-SS <36) | 14 (5.6%) | 5 (4.8%) | 2 (3.0%) |  |
| FSS-ICU at ICU discharge | 26 (20–32) | 28 (21–33) | 28 (21–33) | 0.59 |
| Inability to walk ^a^ | 78 (31.1%) | 35 (33.7%) | 22 (33.3%) | 0.87 |

Definition of abbreviations: COVID-19 = coronavirus disease; ICU = intensive care unit; ARDS = acute respiratory distress syndrome; CFS = Clinical Frailty Scale; MRC-SS = Medical Research Council Sum Score; ICU-AW = Intensive Care Unit Acquired Weakness; FSS-ICU = Functional Status Score for the Intensive Care Unit.

Data are median (quartile 1–quartile 3) or n (%). Percentages may not total 100 because of overlaying or rounding.

^a^ Defined as FSS-ICU walking item <2 points.
