# Supplementary material for: Six-month post-intensive care outcomes during high and low bed occupancy due to the COVID-19 pandemic: A multicenter prospective cohort study
Source: PLoS One. 2023 Nov 16;18(11):e0294631. doi: 10.1371/journal.pone.0294631 (PMC10653414; doi:10.1371/journal.pone.0294631)
Supplement: S4 Table — (DOCX) [file pone.0294631.s005.docx]

**S4 Table. Functional outcomes and employment status at intensive care unit discharge of patients assessed at 3 and 6 months and those lost-to-follow-up**

|  | **Assessed ICU discharge (*n=*252)** | **Lost to follow-up 3 m (n=147)** | **Assessed 3 months (*n=*105)** | ***p-v*alue** | **Lost to follow-up (n=185)** | **Assessed 6 months (*n=*67)** | ***p-v*alue** |
| --- | --- | --- | --- | --- | --- | --- | --- |
| WHODAS–Standardized disability level, % | 27.8 (10.4–48.3) | 27.8 (8.3–50.8) | 28.1 (12.5–45.8) | 0.71 | 27.8 (10.4–47.2) | 28.1 (12.5–50) | 0.70 |
| WHODAS–Total score | 73 (49–101) | 72 (48–102) | 75 (52–99) | 0.71 | 73 (48–101) | 75 (51–105) | 0.71 |
| Understanding & Communicating | 20.8 (4.2–41.7) | 20.8 (4.2–41.7) | 25 (4.2–41.7) | 0.55 | 20.8 (4.2–41.7) | 25 (4.2–45.8) | 0.51 |
| Mobility | 25 (0–60) | 25 (0–75) | 25 (5–55) | 0.58 | 25 (0–65) | 25 (5–55) | 0.47 |
| Self-care | 12.5 (0–50) | 6.25 (0–50) | 12.5 (0–43.8) | 0.79 | 12.5 (0–50) | 12.5 (0–56.3) | 0.73 |
| Getting along with people | 12.5 (0–35) | 10 (0–35) | 15 (0–35) | 0.70 | 10 (0–35) | 20 (0–40) | 0.36 |
| Life Activities: household | 12.5 (0–59.375) | 12.5 (0–62.5) | 12.5 (0–56.3) | 0.70 | 12.5 (0–56.3) | 12.5 (0–75) | 0.52 |
| Life Activities: work or school | 12.5 (0–68.75) | 12.5 (0–75) | 15.625 (0–65.6) | 0.98 | 12.5 (0–62.5) | 12.5 (0–68.8) | 0.94 |
| Participation in society | 37.5 (18.8–62.5) | 37.5 (18.8–62.5) | 37.5 (21.9–62.5) | 0.87 | 37.5 (18.8–62.5) | 37.5 (21.9–62.5) | 0.91 |
| WHODAS–Level of disability |  |  |  | 0.54 |  |  | 0.82 |
| No disability (<5%) | 37 (14.7%) | 21 (14.3%) | 16 (15.2%) |  | 25 (13.5%) | 12 (17.9%) |  |
| Mild disability (5–24%) | 78 (31.0%) | 48 (32.7%) | 30 (28.6%) |  | 59 (31.9%) | 19 (28.4%) |  |
| Moderate disability (25–49%) | 75 (29.8%) | 39 (26.5%) | 36 (34.3%) |  | 56 (30.3%) | 19 (28.4%) |  |
| Severe disability (50–95%) | 62 (24.6%) | 39 (26.5%) | 23 (21.9%) |  | 45 (24.3%) | 17 (25.4%) |  |
| MoCA–Blind | 16 (12–18) | 15 (11–18) | 16 (13–18) | 0.21 | 15 (11–18) | 16 (13–18) | 0.20 |
| Cognitive impairment (<18) | 181 (71.8%) | 108 (73.5%) | 73 (69.5%) | 0.49 | 134 (72.4%) | 47 (70.1%) | 0.72 |
| HADS–depression score | 5 (2–9) | 5 (2–9) | 5 (2–8) | 0.73 | 5 (2–9) | 4 (2–8) | 0.38 |
| Normal (0–7) | 178 (70.6%) | 103 (70.1%) | 75 (71.4%) | 0.97 | 129 (69.7%) | 49 (73.1%) | 0.87 |
| Borderline abnormal (8–10) | 37 (14.7%) | 22 (15.0%) | 15 (14.3%) |  | 28 (15.1%) | 9 (13.4%) |  |
| Abnormal (>11) | 37 (14.7%) | 22 (15.0%) | 15 (14.3%) |  | 28 (15.1%) | 9 (13.4%) |  |
| HADS–anxiety score | 8 (5–12) | 8 (6–13) | 9 (5–11) | 0.45 | 9 (6–13) | 8 (5–11) | 0.26 |
| Normal (0–7) | 107 (42.5%) | 64 (43.5%) | 43 (41.0%) | 0.04 | 78 (42.2%) | 29 (43.3%) | 0.12 |
| Borderline abnormal (8–10) | 49 (19.4%) | 21 (14.3%) | 28 (26.7%) |  | 31 (16.8%) | 18 (26.9%) |  |
| Abnormal (>11) | 96 (38.1%) | 62 (42.2%) | 34 (32.4%) |  | 76 (41.1%) | 20 (29.9%) |  |
| IES-R | 44 (26–56) | 44 (22–56) | 44 (28–55) | 0.47 | 44 (25–56) | 45 (28–55) | 0.58 |
| Normal (0–23) | 52 (20.6%) | 39 (26.5%) | 13 (12.4%) | 0.004 | 42 (22.7%) | 10 (14.9%) | 0.39 |
| Some PTSD symptoms (24–32) | 36 (14.3%) | 13 (8.8%) | 23 (21.9%) |  | 24 (13.0%) | 12 (17.9%) |  |
| Likely diagnosis of PTSD (33–36) | 20 (7.9%) | 12 (8.2%) | 8 (7.6%) |  | 16 (8.6%) | 4 (6.0%) |  |
| PTSD (>36) | 144 (57.1%) | 83 (56.5%) | 61 (58.1%) |  | 103 (55.7%) | 41 (61.2%) |  |

Definition of abbreviations: ICU=Intensive Care Unit; WHODAS = WHO Disability Assessment Schedule; MoCA-blind = Montreal Cognitive Assessment-blind; HADS = Hospital Anxiety and Depression Scale; IES-R = Impact of Event Scale-Revised; PTSD = Post-Traumatic Stress Disorder; EQ-5D-3L = European Quality of Life Health Questionnaire 5 Domains.

Data are median (quartile 1–quartile 3) or n (%). Percentages may not total 100 because of rounding.
