# Supplementary material for: Six-month post-intensive care outcomes during high and low bed occupancy due to the COVID-19 pandemic: A multicenter prospective cohort study
Source: PLoS One. 2023 Nov 16;18(11):e0294631. doi: 10.1371/journal.pone.0294631 (PMC10653414; doi:10.1371/journal.pone.0294631)
Supplement: S5 Table — (DOCX) [file pone.0294631.s006.docx]

**S5 Table. Health related quality of life and employment status at intensive care unit discharge, 3 months and 6 months follow-up according to bed occupancy.**

|  | **ICU discharge** | | | **3-month follow up** | | | **6-month follow up** | | |
| --- | --- | --- | --- | --- | --- | --- | --- | --- | --- |
|  | **Low bed occupancy (*n=*103)** | **High bed occupancy (*n=*149)** | ***p-value*** | **Low bed occupancy (*n=*50)** | **High bed occupancy (*n=*55)** | ***p-v*alue** | **Low bed occupancy (*n=*37)** | **High bed occupancy (*n=*30)** | ***p-v*alue** |
| Problems with mobility | - | - | - | 21 (42%) | 17 (33%) | 0.33 | 12 (33%) | 9 (31%) | 0.84 |
| Problems with personal care | - | - | - | 11(22%) | 3 (6%) | 0.01 | 8 (23%) | 1 (3%) | 0.03 |
| Problems with usual activities | - | - | - | 19 (38%) | 18 (33%) | 0.62 | 16 (43%) | 7 (24%) | 0.11 |
| Problems with pain/discomfort | - | - | - | 29 (58%) | 31 (58%) | 0.96 | 24 (65%) | 14 (48%) | 0.18 |
| Problems with anxiety/ depression | - | - | - | 24 (48%) | 25 (46%) | 0.86 | 16 (43%) | 13 (45%) | 0.90 |
| Baseline employment status |  |  | <0.001 |  |  | 0.01 |  |  | 0.05 |
| Employed–Full Time | 40 (38.8%) | 107 (71.8%) |  | 23 (46%) | 42 (76%) |  | 16 (43%) | 23 (77%) |  |
| Employed–Part Time | 16 (15.5%) | 14 (9.4%) |  | 9 (18%) | 3 (5%) |  | 6 (16%) | 2 (7%) |  |
| Unemployed | 26 (25.2%) | 11 (7.4%) |  | 11 (22%) | 5 (9%) |  | 8 (22%) | 3 (10%) |  |
| Retired | 21 (20.4%) | 17 (11.4%) |  | 7 (14%) | 5 (9%) |  | 7 (19%) | 2 (7%) |  |
| Current employment status | - | - | - |  |  | 0.26 |  |  | 0.52 |
| Employed–Full Time | - | - | - | 16 (32%) | 27 (49%) |  | 17 (46%) | 18 (62%) |  |
| Employed–Part Time | - | - | - | 8 (16%) | 8 (15%) |  | 3 (8%) | 3 (10%) |  |
| Unemployed | - | - | - | 17 (34%) | 14 (25%) |  | 9 (24%) | 4 (14%) |  |
| Retired | - | - | - | 9 (18%) | 5 (9%) |  | 8 (22%) | 4 (14%) |  |
| No answer | - | - | - | 0 (0%) | 1 (2%) |  | - | - | - |
| Change of employment | - | - | - | 38 (76%) | 33 (61%) | 0.20 | 21 (57%) | 20 (67%) | 0.37 |
| Type of employment change | - | - | - |  |  | 0.05 |  |  | 0.08 |
| Same job–fewer hours | - | - | - | 15 (39%) | 19 (58%) |  | 5 (24%) | 10 (50%) |  |
| Different job–same hours | - | - | - | 0 (0%) | 0 (0%) |  | 3 (14%) | 4 (20%) |  |
| Different job–fewer hours | - | - | - | 3 (8%) | 1 (3%) |  | 4 (19%) | 0 (0%) |  |
| Unemployed/Studying | - | - | - | 1 (3%) | 4 (12%) |  | 5 (24%) | 1 (5%) |  |
| Unemployed/ Stopped studying | - | - | - | 19 (50%) | 9 (27%) |  | 4 (19%) | 5 (25%) |  |

Definition of abbreviations: WHODAS = WHO Disability Assessment Schedule; MoCA-blind = Montreal Cognitive Assessment-blind; HADS = Hospital Anxiety and Depression Scale; IES-R = Impact of Event Scale-Revised; PTSD = Post-Traumatic Stress Disorder.

Data are median (quartile 1–quartile 3) or n (%). Percentages may not total 100 because of rounding.
