# Supplementary material for: Six-month post-intensive care outcomes during high and low bed occupancy due to the COVID-19 pandemic: A multicenter prospective cohort study
Source: PLoS One. 2023 Nov 16;18(11):e0294631. doi: 10.1371/journal.pone.0294631 (PMC10653414; doi:10.1371/journal.pone.0294631)
Supplement: S6 Table — (DOCX) [file pone.0294631.s007.docx]

**S6 Table. Functional outcomes and employment status at intensive care unit discharge, 3 months and 6 months follow-up according to COVID-19 infection.**

|  | **ICU discharge** | | | **3-month follow up** | | | **6-month follow up** | | |
| --- | --- | --- | --- | --- | --- | --- | --- | --- | --- |
|  | **Non COVID-19 (*n=*60)** | **COVID-19 (*n=*192)** | ***p-value*** | **Non COVID-19 (*n=*22)** | **COVID-19 (*n=*83)** | ***p-value*** | **Non COVID-19 (*n=*15)** | **COVID-19 (*n=*52)** | ***p-v*alue** |
| WHODAS–Standardized disability level, % | 31.3 (14.0–48.3) | 26 (8.0–49.2) | 0.18 | 8.5 (4.7–20.5) | 12.5 (4.2–28.6) | 0.73 | 8.6 (0.8–25.0) | 9.8 (2.9–21.4) | 0.52 |
| WHODAS–Total score | 76.5 (54.5–97.5) | 68.5 (47–102) | 0.43 | 38.5 (28–48) | 44 (30–54) | 0.26 | 37 (25–49) | 36.5 (31.5–46) | 0.41 |
| Understanding & communicating | 25 (4.2–41.7) | 20.8 (4.2–41.7) | 0.24 | 8.3 (4.2–20.8) | 8.3 (0–29.2) | 0.91 | 4.2 (0–45.8) | 12.5 (0–29.2) | 0.45 |
| Mobility | 30 (10–55) | 25 (0–65) | 0.40 | 5 (0–30) | 15 (0–40) | 0.24 | 5 (0–30) | 5 (0–40) | 0.81 |
| Self-Care | 12.5 (0–50) | 9.4 (0–46.9) | 0.78 | 0 (0–6.3) | 0 (0–12.5) | 0.63 | 0 (0–6.3) | 0 (0–0) | 0.17 |
| Getting along with people | 20 (5–37.5) | 10 (0–35) | 0.16 | 5 (0–15) | 0 (0–15) | 0.42 | 0 (0–10) | 0 (0–10) | 0.83 |
| Life Activities: household | 18.8 (0–53.1) | 12.5 (0–62.5) | 0.66 | 6.3 (0–12.5) | 6.3 (0–25) | 0.77 | 0 (0–18.8) | 0 (0–31.3) | 0.76 |
| Life Activities: work or school | 12.5 (0–56.3) | 12.5 (0–75) | 0.62 | 25 (6.3–50) | 12.5 (0–25) | 0.19 | 0 (0–25) | 0 (0–31.3) | 0.84 |
| Participation in society | 43.8 (26.6–65.6) | 37.5 (17.2–59.4) | 0.13 | 18.8 (9.4–21.9) | 20.3 (15.6–39.1) | 0.72 | 15.6 (3.1–25) | 9.4 (3.1–37.5) | 0.89 |
| WHODAS–Level of disability |  |  | 0.018 |  |  | 0.80 |  |  | 0.41 |
| No disability (<5%) | 3 (5.0%) | 34 (17.7%) |  | 7 (32%) | 26 (31%) |  | 7 (47%) | 20 (38%) |  |
| Mild disability (5–24%) | 17 (28.3%) | 61 (31.8%) |  | 10 (45%) | 30 (36%) |  | 4 (27%) | 23 (44%) |  |
| Moderate disability (25–49%) | 26 (43.3%) | 49 (25.5%) |  | 4 (18%) | 23 (28%) |  | 4 (27%) | 7 (13%) |  |
| Severe disability (50–95%) | 14 (23.3%) | 48 (25.0%) |  | 1 (5%) | 4 (5%) |  | 0 (0%) | 2 (4%) |  |
| MoCA–Blind | 14.5 (11–17) | 16 (12–18) | 0.09 | 18 (14–19) | 19 (16–21) | 0.06 | 20 (18–22) | 21 (18–22) | 0.48 |
| Cognitive impairment (<18) | 47 (78.3%) | 134 (69.8%) | 0.20 | 10 (45%) | 28 (34%) | 0.31 | 2 (13%) | 9 (18%) | 0.69 |
| HADS–depression score | 6 (3–10) | 5 (2–8) | 0.16 | 7 (5–9) | 6 (5–10) | 0.69 | 7 (6–11) | 7 (5–9) | 0.46 |
| Normal (0–7) | 39 (65.0%) | 139 (72.4%) | 0.22 | 13 (59%) | 47 (57%) | 0.86 | 9 (60%) | 32 (62%) | 0.38 |
| Borderline abnormal (8–10) | 13 (21.7%) | 24 (12.5%) |  | 4 (18%) | 19 (23%) |  | 2 (13%) | 13 (25%) |  |
| Abnormal (>11) | 8 (13.3%) | 29 (15.1%) |  | 5 (23%) | 16 (20%) |  | 4 (27%) | 7 (13%) |  |
| HADS–anxiety score | 10 (7–12.5) | 8 (5–12) | 0.14 | 6 (5–8) | 6 (4–10) | 0.94 | 4 (3–9) | 7 (3–10) | 0.52 |
| Normal (0–7) | 21 (35.0%) | 86 (44.8%) | 0.27 | 14 (64%) | 51 (62%) | 0.99 | 11 (73%) | 34 (65%) | 0.79 |
| Borderline abnormal (8–10) | 11 (18.3%) | 38 (19.8%) |  | 4 (18%) | 15 (18%) |  | 2 (13%) | 7 (13%) |  |
| Abnormal (>11) | 28 (46.7%) | 68 (35.4%) |  | 4 (18%) | 16 (20%) |  | 2 (13%) | 11 (21%) |  |
| IES-R | 46.5 (27–56.5) | 43 (25.5–55) | 0.30 | 19 (8–39) | 21 (8–40) | 0.84 | 10 (6–45) | 20.5 (6–40.5) | 0.78 |
| Normal (0–23) | 12 (20.0%) | 40 (20.8%) | 0.22 | 12 (57%) | 44 (53%) | 0.88 | 8 (53%) | 29 (56%) | 0.42 |
| Some PTSD symptoms (24–32) | 6 (10.0%) | 30 (15.6%) |  | 2 (10%) | 10 (12%) |  | 0 (0%) | 6 (12%) |  |
| Likely diagnosis of PTSD (33–36) | 2 (3.3%) | 18 (9.4%) |  | 1 (5%) | 8 (10%) |  | 2 (13%) | 3 (6%) |  |
| PTSD (>36) | 40 (66.7%) | 104 (54.2%) |  | 6 (29%) | 21 (25%) |  | 5 (33%) | 14 (27%) |  |
| EQ-5D-3L |  |  |  |  |  |  |  |  |  |
| Problems with mobility | - | - |  | 9 (43%) | 29 (36%) | 0.55 | 4 (29%) | 17 (33%) | 1.0 |
| Problems with personal care | - | - |  | 2 (9%) | 12 (15%) | 0.50 | 2 (13%) | 7 (14%) | 1.0 |
| Problems with usual activities | - | - |  | 4 (18%) | 33 (40%) | 0.06 | 6 (40%) | 17 (33%) | 0.76 |
| Problems with pain/discomfort | - | - |  | 12 (55%) | 48 (59%) | 0.69 | 8 (53%) | 30 (59%) | 0.77 |
| Problems with anxiety/ depression | - | - |  | 12 (55%) | 37 (45%) | 0.43 | 6 (40%) | 23 (45%) | 0.78 |
| Baseline employment status |  |  | <0.001 |  |  | 0.004 |  |  | 0.04 |
| Employed–Full Time | 20 (33.3%) | 127 (66.1%) |  | 7 (32%) | 58 (70%) |  | 5 (33%) | 34 (65%) |  |
| Employed–Part Time | 10 (16.7%) | 20 (10.4%) |  | 6 (27%) | 6 (7%) |  | 4 (27%) | 4 (8%) |  |
| Unemployed | 13 (21.7%) | 24 (12.5%) |  | 4 (18%) | 12 (14%) |  | 2 (13%) | 9 (17%) |  |
| Retired | 17 (28.3%) | 21 (10.9%) |  | 5 (23%) | 7 (8%) |  | 4 (27%) | 5 (10%) |  |
| Current employment status | - | - |  |  |  | 0.59 |  |  | 1.0 |
| Employed–Full Time | - | - |  | 8 (36%) | 35 (42%) |  | 8 (53%) | 27 (53%) |  |
| Employed–Part Time | - | - |  | 2 (9%) | 14 (17%) |  | 1 (7%) | 5 (10%) |  |
| Unemployed | - | - |  | 7 (32%) | 24 (29%) |  | 3 (20%) | 10 (20%) |  |
| Retired | - | - |  | 5 (23%) | 9 (112%) |  | 3 (20%) | 9 (18%) |  |
| No answer | - | - |  | 0 (0%) | 1 (1%) |  | - | - |  |
| Change of employment |  | - |  | 12 (55%) | 59 (72%) | 0.15 | 5 (33%) | 36 (69%) | 0.025 |
| Type of employment change | - | - |  |  |  | 0.006 |  |  | 0.37 |
| Same job–fewer hours | - | - |  | 5 (42%) | 29 (49%) |  | 2 (40%) | 13 (36%) |  |
| Different job–same hours | - | - |  | 0 (0%) | 0 (0%) |  | 1 (20%) | 6 (17%) |  |
| Different job–fewer hours | - | - |  | 1 (8%) | 3 (5%) |  | 0 (0%) | 4 (11%) |  |
| Unemployed/Studying | - | - |  | 4 (33%) | 1 (2%) |  | 1 (20%) | 5 (14%) |  |
| Unemployed/ Stopped studying | - | - |  | 2 (17%) | 26 (44%) |  | 1 (20%) | 8 (22%) |  |
| No answer | - | - |  | 0 (0%) | 0 (0%) |  | - | - |  |

Definition of abbreviations: COVID-19 = coronavirus disease; WHODAS = WHO Disability Assessment Schedule; MoCA-blind = Montreal Cognitive Assessment-blind; HADS = Hospital Anxiety and Depression Scale; IES-R = Impact of Event Scale-Revised; PTSD = Post-Traumatic Stress Disorder.

Data are median (quartile 1–quartile 3) or n (%). Percentages may not total 100 because of rounding.
