# Supplementary material for: Six-month post-intensive care outcomes during high and low bed occupancy due to the COVID-19 pandemic: A multicenter prospective cohort study
Source: PLoS One. 2023 Nov 16;18(11):e0294631. doi: 10.1371/journal.pone.0294631 (PMC10653414; doi:10.1371/journal.pone.0294631)
Supplement: S7 Table — (DOCX) [file pone.0294631.s008.docx]

**S7 Table. Functional outcomes at intensive care unit discharge according to bed occupancy and COVID-19 infection.**

|  | Non COVID-19 | | | COVID-19 | | |
| --- | --- | --- | --- | --- | --- | --- |
|  | Low bed occupancy (*n=*37) | High bed occupancy (*n=*23) | *p-value* | Low bed occupancy (*n=*66) | High bed occupancy (*n=*126) | *p-value* |
| WHODAS–Standardized disability level, % | 31 (14–44) | 31 (15–50) | 0.72 | 29 (8.3–61) | 22.6 (7.8–41) | 0.097 |
| WHODAS–Total score | 76 (51–96) | 77 (58–106) | 0.58 | 76 (48–123) | 68 (47–94) | 0.17 |
| Understanding & Communicating | 25 (4.2–33.3) | 25 (4.2–45.8) | 0.31 | 23 (4–45.8) | 14.6 (0–33.3) | 0.13 |
| Mobility | 25 (10–50) | 40 (10–60) | 0.57 | 35 (0–85) | 20 (0–45) | 0.037 |
| Self Care | 12.5 (0–43.8) | 6.3 (0–68.8) | 0.91 | 22 (0–68.8) | 6.3 (0–37.5) | 0.32 |
| Getting along with people | 15 (5–35) | 20 (5–45) | 0.71 | 20 (0–40) | 5 (0–25) | 0.037 |
| Life Activities: household | 12.5 (0–50) | 31.3 (0–68.8) | 0.49 | 18.8 (0–81.3) | 12.5 (0–56.3) | 0.25 |
| Life Activities: work or school | 43.8 (6.3–75) | 9.4 (0–50) | 0.3 | 12.5 (0–75) | 12.5 (0–68.8) | 0.95 |
| Participation in society | 50 (31–68.8) | 37.5 (25–62.5) | 0.49 | 39 (18.8–68.8) | 34 (15.6–56) | 0.2 |
| WHODAS–Level of disability |  |  | 0.97 |  |  | 0.047 |
| No disability (<5%) | 2 (5.4%) | 1 (4.3%) |  | 12 (18.2%) | 22 (17.5%) |  |
| Mild disability (5–24%) | 11 (29.7%) | 6 (26.1%) |  | 18 (27.3%) | 43 (34.1%) |  |
| Moderate disability (25–49%) | 16 (43.2%) | 10 (43.5%) |  | 12 (18.2%) | 37 (29.4%) |  |
| Severe disability (50–95%) | 8 (21.6%) | 6 (26.1%) |  | 24 (36.4%) | 24 (19.0%) |  |
| MoCA–Blind | 15 (11–17) | 14 (10–17) | 0.72 | 16 (11–18) | 16 (13–18) | 0.36 |
| Cognitive impairment (<18) | 29 (78.4%) | 18 (78.3%) | 0.99 | 46 (69.7%) | 88 (69.8%) | 0.98 |
| HADS–depression score | 5 (2–8) | 7 (4–10) | 0.05 | 5.5 (3–10) | 4 (2–7) | 0.058 |
| Normal (0–7) | 27 (73.0%) | 12 (52.2%) | 0.26 | 42 (63.6%) | 97 (77.0%) | 0.08 |
| Borderline abnormal (8–10) | 6 (16.2%) | 7 (30.4%) |  | 9 (13.6%) | 15 (11.9%) |  |
| Abnormal (>11) | 4 (10.8%) | 4 (17.4%) |  | 15 (22.7%) | 14 (11.1%) |  |
| HADS–anxiety score | 9 (7–12) | 11 (7–13) | 0.37 | 8.5 (4–12) | 8 (5–12) | 0.94 |
| Normal (0–7) | 13 (35.1%) | 8 (34.8%) | 0.26 | 29 (43.9%) | 57 (45.2%) | 0.94 |
| Borderline abnormal (8–10) | 9 (24.3%) | 2 (8.7%) |  | 14 (21.2%) | 24 (19.0%) |  |
| Abnormal (>11) | 15 (40.5%) | 13 (56.5%) |  | 23 (34.8%) | 45 (35.7%) |  |
| IES-R | 50 (26–57) | 46 (29–56) | 0.9 | 44 (29–56) | 40 (25–55) | 0.4 |
| Normal (0–23) | 8 (21.6%) | 4 (17.4%) | 0.63 | 12 (18.2%) | 28 (22.2%) | 0.51 |
| Some PTSD symptoms (24–32) | 4 (10.8%) | 2 (8.7%) |  | 10 (15.2%) | 20 (15.9%) |  |
| Likely diagnosis of PTSD (33–36) | 2 (5.4%) | 0 (0.0%) |  | 4 (6.1%) | 14 (11.1%) |  |
| PTSD (>36) | 23 (62.2%) | 17 (73.9%) |  | 40 (60.6%) | 64 (50.8%) |  |

Definition of abbreviations: COVID-19 = coronavirus disease; WHODAS = WHO Disability Assessment Schedule; MoCA-blind = Montreal Cognitive Assessment-blind; HADS = Hospital Anxiety and Depression Scale; IES-R = Impact of Event Scale-Revised; PTSD = Post-Traumatic Stress Disorder.

Data are median (quartile 1–quartile 3) or n (%). Percentages may not total 100 because of rounding
