# Supplementary material for: Six-month post-intensive care outcomes during high and low bed occupancy due to the COVID-19 pandemic: A multicenter prospective cohort study
Source: PLoS One. 2023 Nov 16;18(11):e0294631. doi: 10.1371/journal.pone.0294631 (PMC10653414; doi:10.1371/journal.pone.0294631)
Supplement: S8 Table — (DOCX) [file pone.0294631.s009.docx]

**S8 Table. Functional outcomes and employment status at 3 months and 6 months follow-up according to COVID-19 infection and bed occupancy.**

|  | **3-month follow up** | | | | | | **6-month follow up** | | | | | |
| --- | --- | --- | --- | --- | --- | --- | --- | --- | --- | --- | --- | --- |
|  | **non COVID-19** | | | **COVID-19** | | | **non COVID-19** | | | **COVID-19** | | |
|  | **Low occupancy (*n=12*)** | **High occupancy (n=10)** | ***p-value*** | **Low occupancy (*n=38*)** | **High occupancy (n=45)** | ***p-value*** | **Low occupancy (*n=11*)** | **High occupancy (n=4)** | ***p-value*** | **Low occupancy (*n=26*)** | **High occupancy (n=26)** | ***p-value*** |
| WHODAS–Standardized disability level, % | 6.9 (4.7–19.2) | 16.7 (2.2–25) | 0.69 | 14.8 (4.7–27) | 9.4 (3.6–29) | 0.99 | 13.5 (0.9–26) | 1.3 (0.4–13) | 0.29 | 13.6 (2–23) | 5.8 (3.6–18) | 0.35 |
| WHODAS, Total score | 35.5 (29–41) | 40.5 (25–53) | 0.57 | 42.5 (35–53) | 44 (28–55) | 0.99 | 37 (25–49) | 30 (25–49) | 0.65 | 39 (30–52) | 35.5 (32–44) | 0.55 |
| Understanding & communicating | 8.3 (2.1–17) | 12.5 (4.2–42) | 0.40 | 8.3 (0–25) | 8.3 (4–38) | 0.24 | 8.3 (0–46) | 2.1 (0–48) | 0.89 | 14.6 (0–29) | 12.5 (0–29) | 0.92 |
| Mobility | 5 (0–22.5) | 2.5 (0–50) | 0.97 | 20 (0–40) | 15 (0–40) | 0.94 | 15 (0–30) | 0 (0–2.5) | 0.067 | 5 (0–40) | 7.5 (0–45) | 0.96 |
| Self-Care | 0 (0–3.1) | 0 (0–6.25) | 0.67 | 0 (0–12.5) | 0 (0–12.5) | 0.52 | 0 (0–18.8) | 0 (0–0) | 0.12 | 0 (0–0) | 0 (0–0) | 0.15 |
| Getting along with people | 5 (0–17.5) | 5 (0–10) | 0.81 | 0 (0–15) | 5 (0–10) | 0.46 | 0 (0–20) | 0 (0–5) | 0.46 | 0 (0–10) | 0 (0–15) | 0.48 |
| Life Activities: household | 6.3 (0–12.5) | 6.3 (0–44) | 0.81 | 6.3 (0–25) | 6.25 (0–25) | 0.70 | 12.5 (0–31.3) | 0 (0–0) | 0.047 | 9.4 (0–38) | 0 (0–19) | 0.13 |
| Life Activities: work or school | 18.8 (0–31) | 50 (34–75) | 0.11 | 12.5 (0–25) | 22 (0–50) | 0.41 | 12.5 (0–25) | 0 (0–0) | 0.40 | 25 (6.25–50) | 0 (0–0) | 0.007 |
| Participation in society | 18.8 (9–72) | 15.6 (9–21.9) | 0.77 | 15.6 (16–41) | 28 (19–38) | 0.24 | 15.6 (15–16) | 14 (3–25) | 1.00 | 31.3 (6–38) | 6.3 (0–9) | 0.043 |
| WHODAS–Level of disability |  |  | 0.71 |  |  | 0.99 |  |  | 0.30 |  |  | 0.94 |
| No disability (<5%) | 4 (33%) | 3 (30%) |  | 12 (32%) | 14 (31%) |  | 4 (36%) | 3 (75%) |  | 9 (35%) | 11 (42%) |  |
| Mild disability (5–24%) | 6 (50%) | 4 (40%) |  | 14 (37%) | 16 (36%) |  | 4 (36%) | 0 (0%) |  | 12 (46%) | 11 (42%) |  |
| Moderate disability (25–49%) | 2 (17%) | 2 (20%) |  | 10 (26%) | 13 (29%) |  | 3 (27%) | 1 (25%) |  | 4 (15%) | 3 (12%) |  |
| Severe disability (50–95%) | 0 (0%) | 1 (10%) |  | 2 (5%) | 2 (4%) |  |  |  |  | 1 (4%) | 1 (4%) |  |
| MoCA–Blind | 18 (13–19) | 17.5 (15–21) | 0.59 | 18 (15–20) | 20 (17.5–21) | 0.047 | 20 (18–22) | 20.5 (17.5–22) | 0.74 | 20.5 (18–21.5) | 21 (19–22) | 0.15 |
| Cognitive impairment (<18) | 5 (42%) | 5 (50%) | 0.70 | 17 (45%) | 11 (25%) | 0.060 | 1 (9%) | 1 (25%) | 0.42 | 5 (20%) | 4 (15%) | 0.67 |
| HADS–depression score | 7 (6–8.5) | 6 (5–11) | 0.57 | 7 (5–10) | 6 (5–9.5) | 0.47 | 8 (5–11) | 6.5 (6–7) | 0.60 | 7 (5–9) | 7 (5–8) | 0.74 |
| Normal (0–7) | 7 (58%) | 6 (60%) | 0.58 | 20 (53%) | 27 (61%) | 0.51 | 5 (45%) | 4 (100%) | 0.16 | 15 (58%) | 17 (65%) | 0.62 |
| Borderline abnormal (8–10) | 3 (25%) | 1 (10%) |  | 11 (29%) | 8 (18%) |  | 2 (18%) | 0 (0%) |  | 8 (31%) | 5 (19%) |  |
| Abnormal (>11) | 2 (17%) | 3 (30%) |  | 7 (18%) | 9 (20%) |  | 4 (36%) | 0 (0%) |  | 3 (12%) | 4 (15%) |  |
| HADS–anxiety score | 7 (3.5–10) | 6 (5–7) | 0.87 | 6 (4–9) | 6 (4.5–10) | 0.56 | 4 (3–10) | 5 (3–6) | 0.51 | 7 (3–10) | 6.5 (3–10) | 0.80 |
| Normal (0–7) | 6 (50%) | 8 (80%) | 0.35 | 25 (66%) | 26 (59%) | 0.81 | 7 (64%) | 4 (100%) | 0.37 | 17 (65%) | 17 (65%) | 0.89 |
| Borderline abnormal (8–10) | 3 (25%) | 1 (10%) |  | 6 (16%) | 9 (20%) |  | 2 (18%) | 0 (0%) |  | 4 (15%) | 3 (12%) |  |
| Abnormal (>11) | 3 (25%) | 1 (10%) |  | 7 (18%) | 9 (20%) |  | 2 (18%) | 0 (0%) |  | 5 (19%) | 6 (23%) |  |
| IES-R | 19 (6–33) | 21 (10–54) | 0.25 | 20.5 (7–43) | 21 (12–36) | 0.82 | 38 (7–46) | 7.5 (4.5–24) | 0.15 | 21 (4–39) | 17.5 (7–42) | 0.96 |
| Normal (0–23) | 7 (64%) | 5 (50%) | 0.58 | 20 (53%) | 24 (53%) | 0.87 | 5 (45%) | 3 (75%) | 0.24 | 15 (58%) | 14 (54%) | 0.79 |
| Some PTSD symptoms (24–32) | 1 (9%) | 1 (10%) |  | 4 (11%) | 6 (13%) |  |  |  |  | 2 (8%) | 4 (15%) |  |
| Likely diagnosis of PTSD (33–36) | 1 (9%) | 0 (0%) |  | 3 (8%) | 5 (11%) |  | 1 (9%) | 1 (25%) |  | 2 (8%) | 1 (4%) |  |
| PTSD (>36) | 2 (18%) | 4 (40%) |  | 11 (29%) | 10 (22%) |  | 5 (45%) | 0 (0%) |  | 7 (27%) | 7 (27%) |  |
| EQ-5D-3L | 0.7 (0.5–0.8) | 0.8 (0.7–0.8) | 0.62 | 0.7 (0.5–0.8) | 0.7 (0.6–0.8) | 0.45 | 0.8 (0.6–0.8) | 0.9 (0.6–1) | 0.25 | 0.7 (0.4–0.8) | 0.8 (0.6–1) | 0.17 |
| Problems with mobility | 6 (50%) | 3 (33%) | 0.66 | 15 (39%) | 14 (33%) | 0.64 | 3 (30%) | 1 (25%) | 1.00 | 9 (35%) | 8 (32%) | 1.00 |
| Problems with personal care | 1 (8%) | 1 (10%) | 1.00 | 10 (26%) | 2 (5%) | 0.010 | 2 (18%) | 0 (0%) | 1.00 | 6 (25%) | 1 (4%) | 0.049 |
| Problems with usual activities | 3 (25%) | 1 (10%) | 0.59 | 16 (42%) | 17 (39%) | 0.82 | 6 (55%) | 0 (0%) | 0.10 | 10 (38%) | 7 (28%) | 0.56 |
| Problems with pain/discomfort | 6 (50%) | 6 (60%) | 0.69 | 23 (61%) | 25 (58%) | 1.00 | 7 (64%) | 1 (25%) | 0.28 | 17 (65%) | 13 (52%) | 0.40 |
| Problems with anxiety/ depression | 7 (58%) | 5 (50%) | 1.00 | 17 (45%) | 20 (45%) | 1.00 | 4 (36%) | 2 (50%) | 1.00 | 12 (46%) | 11 (44%) | 1.00 |
| Baseline employment status |  |  | 0.84 |  |  | 0.009 |  |  | 1.00 |  |  | 0.15 |
| Employed–Full Time | 3 (25%) | 4 (40%) |  | 20 (53%) | 38 (84%) |  | 3 (27%) | 2 (50%) |  | 13 (50%) | 21 (81%) |  |
| Employed–Part Time | 4 (33%) | 2 (20%) |  | 5 (13%) | 1 (2%) |  | 3 (27%) | 1 (25%) |  | 3 (12%) | 1 (4%) |  |
| Unemployed | 2 (17%) | 2 (20%) |  | 9 (24%) | 3 (7%) |  | 2 (18%) | 0 (0%) |  | 6 (23%) | 3 (12%) |  |
| Retired | 3 (25%) | 2 (20%) |  | 4 (11%) | 3 (7%) |  | 3 (27%) | 1 (25%) |  | 4 (15%) | 1 (4%) |  |
| Current employment status |  |  | 0.14 |  |  | 0.70 |  |  | 0.82 |  |  | 0.64 |
| Employed–Full Time | 2 (17%) | 6 (60%) |  | 14 (37%) | 21 (47%) |  | 5 (45%) | 3 (75%) |  | 12 (46%) | 15 (60%) |  |
| Employed–Part Time | 2 (17%) | 0 (0%) |  | 6 (16%) | 8 (18%) |  | 1 (9%) | 0 (0%) |  | 2 (8%) | 3 (12%) |  |
| Unemployed | 4 (33%) | 3 (30%) |  | 13 (34%) | 11 (24%) |  | 3 (27%) | 0 (0%) |  | 6 (23%) | 4 (16%) |  |
| Retired | 4 (33%) | 1 (10%) |  | 5 (13%) | 4 (9%) |  | 2 (18%) | 1 (25%) |  | 6 (23%) | 3 (12%) |  |
| No answer |  |  |  | 0 (0%) | 1 (2%) |  |  |  |  |  |  |  |
| Change of employment | 5 (42%) | 7 (70%) | 0.39 | 33 (87%) | 26 (58%) | 0.007 | 3 (27%) | 2 (50%) | 0.56 | 18 (69%) | 18 (69%) | 1.00 |
| Type of employment change |  |  | 0.50 |  |  | 0.006 |  |  | 0.41 |  |  | 0.16 |
| Same job–fewer hours | 2 (17%) | 3 (30%) |  | 13 (34%) | 16 (36%) |  | 1 (9%) | 1 (25%) |  | 4 (15%) | 9 (35%) |  |
| Different job–same hours |  |  |  |  |  |  |  |  |  | 3 (12%) | 3 (12%) |  |
| Different job–fewer hours | 1 (8%) | 0 (0%) |  | 2 (5%) | 1 (2%) |  | 0 (0%) | 1 (25%) |  | 4 (15%) | 0 (0%) |  |
| Unemployed/Studying | 1 (8%) | 3 (30%) |  | 0 (0%) | 1 (2%) |  | 1 (9%) | 0 (0%) |  | 4 (15%) | 1 (4%) |  |
| Unemployed/ Stopped studying | 1 (8%) | 1 (10%) |  | 18 (47%) | 8 (18%) |  | 1 (9%) | 0 (0%) |  | 3 (12%) | 5 (19%) |  |
| No answer |  |  |  | 0 (0%) | 1 (2%) |  |  |  |  | 0 (0%) | 1 (4%) |  |

Definition of abbreviations: COVID-19 = coronavirus disease; WHODAS = WHO Disability Assessment Schedule; MoCA-blind = Montreal Cognitive Assessment-blind; HADS = Hospital Anxiety and Depression Scale; IES-R = Impact of Event Scale-Revised; PTSD = Post-Traumatic Stress Disorder.

Data are median (quartile 1–quartile 3) or n (%). Percentages may not total 100 because of rounding.
