# Supplementary material for: Six-month post-intensive care outcomes during high and low bed occupancy due to the COVID-19 pandemic: A multicenter prospective cohort study
Source: PLoS One. 2023 Nov 16;18(11):e0294631. doi: 10.1371/journal.pone.0294631 (PMC10653414; doi:10.1371/journal.pone.0294631)
Supplement: S9 Table — (DOCX) [file pone.0294631.s010.docx]

**S9 Table. IMPACCT COVID-19 study group sites and evaluators.**

| **City** | **Site Name** | **Site evaluators** | **Site coordinators** |
| --- | --- | --- | --- |
| Santiago | Clínica Alemana de Santiago | Agustín Camus-Molina | Agustín Camus-Molina |
|  |  | Cristóbal Enríquez Selaive | Felipe González-Seguel |
|  |  | Felipe González-Seguel |  |
|  |  | Fernanda Galleguillos-Cabello |  |
|  |  | Gabriela Meza |  |
|  |  | Josefina León Urcelay |  |
|  |  | Macarena Leiva-Corvalán |  |
|  |  | Nadine Aranis Seguic |  |
|  |  | Sebastián Sainte-marie |  |
| Santiago | Clínica BUPA | Catherine Verdugo | Javiera Aguilera Scarpati |
|  |  | Diego Orellana Navarro | Joaquin Olave Cerna |
|  |  | Javiera Aguilera Scarpati |  |
|  |  | Joaquin Olave Cerna |  |
|  |  | Joshua Duran |  |
|  |  | Rodrigo Flores Cartes |  |
| Santiago | Clínica INDISA | Benito Arévalo Pereda | Felipe Muñoz-Muñoz |
|  |  | Felipe Muñoz-Muñoz |  |
|  |  | Juan José Pinto-Concha |  |
|  |  | Maria Paz Fuster Vespremy |  |
|  |  | Natalia Miranda Villegas |  |
|  |  | Nelson Barra Garcés |  |
|  |  | Nicol Jara |  |
|  |  | Nicole Peña |  |
| Santiago | Hospital del Salvador | Agustina Hoffmann | Camilo Cáceres-Parra |
|  |  | Annemarie Vargas | Macarena Leiva-Corvalán |
|  |  | Bernardita Larraín |  |
|  |  | Camilo Cáceres-Parra |  |
|  |  | Constanza Romero |  |
|  |  | Davor Cibilic |  |
|  |  | Diego López Arnello |  |
|  |  | Lissette Cabezas |  |
|  |  | Macarena Leiva-Corvalán |  |
|  |  | Martin Perez Rossi |  |
|  |  | Millaray Cantillano Matus |  |
|  |  | Pedro Vergara |  |
| Santiago | Hospital Metropolitano | Claudia Jara | Fernanda Baus Auil |
|  |  | Claudia Sepúlveda |  |
|  |  | Cynthia Guajardo Inostroza |  |
|  |  | Felipe Cuevas |  |
|  |  | Fernanda Baus Auil |  |
|  |  | Max Ibacache |  |
| Antofagasta | Hospital Regional Dr Leonardo | Constanza Morales | Yerko Villagra-Jofré |
|  | Guzmán de Antofagasta | María Ángel Suarez | Pilar Castro Gaitán |
|  |  | Pilar Castro Gaitán |  |
|  |  | Yerko Villagra Jofré |  |
| Coquimbo | Hospital San Pablo de Coquimbo | Alvaro Wilson Espinoza | Eduardo González Tapia |
|  |  | Diego Melo Villagrán |  |
|  |  | Eduardo González Tapia |  |
|  |  | Karen González Vásquez |  |
|  |  | Magdalena Contardo Cisternas |  |
|  |  | María José Vega Astudillo |  |
|  |  | Sergio Ortiz Gómez |  |
