# Supplementary material for: Six-month post-intensive care outcomes during high and low bed occupancy due to the COVID-19 pandemic: A multicenter prospective cohort study
Source: PLoS One. 2023 Nov 16;18(11):e0294631. doi: 10.1371/journal.pone.0294631 (PMC10653414; doi:10.1371/journal.pone.0294631)
Supplement: Attachment — Submitted filename: Response to Reviewers.docx [file pone.0294631.s012.docx]

**Response Letter**

| **Manuscript Title:** | Six-Month Post-Intensive Care Outcomes During High and Low Bed Occupancy due to the COVID-19 Pandemic: A Multicenter Prospective Cohort Study |
| --- | --- |
| **Manuscript Type:** | Original study |
| **Manuscript ID:** | PONE-D-23-23670 |
| Date: | October 23, 2023 |
| Corresponding Author Name: | Felipe González-Seguel |
| E-mail: | [feligonzalezs@udd.cl](mailto:feligonzalezs@udd.cl) |

Dear Editor and Reviewers:

We would like to thank you for your valuable time and useful contribution. We appreciate the comments you have given us to improve our manuscript. The next section contains the changes that were made using boldface font in the “Manuscript_Track Changes_R1” based on the reviewer and editor’s comments.

**Point by point answer to the Editor and Reviewer:**

**JOURNAL REQUIREMENTS:**

When submitting your revision, we need you to address these additional requirements.

1. Please ensure that your manuscript meets PLOS ONE's style requirements, including those for file naming. The PLOS ONE style templates can be found at

<https://journals.plos.org/plosone/s/file?id=wjVg/PLOSOne_formatting_sample_main_body.pdf> and

<https://journals.plos.org/plosone/s/file?id=ba62/PLOSOne_formatting_sample_title_authors_affiliations.pdf>

**ANSWER**: We edited each section of our manuscript following the PlosOne templates.

2. Thank you for stating the following in the Acknowledgments Section of your manuscript:

“We want to acknowledge the Universidad del Desarrollo for funding this research and each participating site for allowing us to use part of their infrastructure (organized alphabetically): Clínica Alemana de Santiago, Clínica BUPA, Clínica INDISA, Hospital del Salvador, Hospital Metropolitano, Hospital Regional Dr. Leonardo Guzmán de Antofagasta, and Hospital San Pablo de Coquimbo.”

We note that you have provided funding information that is currently declared in your Funding Statement. However, funding information should not appear in the Acknowledgments section or other areas of your manuscript. We will only publish funding information present in the Funding Statement section of the online submission form.

Please remove any funding-related text from the manuscript and let us know how you would like to update your Funding Statement. Currently, your Funding Statement reads as follows:

“IMPACCT COVID-19 study was funded by Universidad del Desarrollo (Grant number 2020-78) and sponsored by the Chilean National Agency for Research and Development (ANID-0772). The funders had no role in the design, collection, analysis of the study, and writing of this manuscript. Received by CM-O.”

Please include your amended statements within your cover letter; we will change the online submission form on your behalf.

**ANSWER**: We have removed funding information from the acknowledgement section of the manuscript. The funding statement will be the following: “IMPACCT COVID-19 study was funded by Universidad del Desarrollo (Grant number 2020-78) and sponsored by the Chilean National Agency for Research and Development (ANID-0772). The funders had no role in the design, collection, analysis of the study, and writing of this manuscript. Received by CM-O.”

3. We note that you have indicated that data from this study are available upon request. PLOS only allows data to be available upon request if there are legal or ethical restrictions on sharing data publicly. For more information on unacceptable data access restrictions, please see <http://journals.plos.org/plosone/s/data-availability#loc-unacceptable-data-access-restrictions>.

In your revised cover letter, please address the following prompts:

a) If there are ethical or legal restrictions on sharing a de-identified data set, please explain them in detail (e.g., data contain potentially sensitive information, data are owned by a third-party organization, etc.) and who has imposed them (e.g., an ethics committee). Please also provide contact information for a data access committee, ethics committee, or other institutional body to which data requests may be sent.

b) If there are no restrictions, please upload the minimal anonymized data set necessary to replicate your study findings as either Supporting Information files or to a stable, public repository and provide us with the relevant URLs, DOIs, or accession numbers. For a list of acceptable repositories, please see <http://journals.plos.org/plosone/s/data-availability#loc-recommended-repositories>.

We will update your Data Availability statement on your behalf to reflect the information you provide.

**ANSWER**: We selected option a), because the consent form approved by our ethics committee (<https://www.udd.cl/comite-de-etica-y-regulatorio/> and <https://www.clinicaalemana.cl/desarrollo-academico-e-investigacion/investigacion>) explicitly states that individual patient data, even anonymized, will only be available to the authors for a period of five years.

The exact wording in the consent form in Spanish says:

*Confidencialidad: Tanto la información otorgada por usted como su información personal será resguardada por un período de 5 años posterior al estudio bajo llave y encriptados. El manejo y almacenamiento de la información se realizará codificada de manera confidencial durante el proceso de investigación, y teniendo acceso único los autores del estudio. La información recolectada será presentada en forma agregada en publicaciones y conferencias científicas.*

*Which means:*

*Confidentiality: both, your personal information and the information provided by you will be kept safe, locked, and encrypted for a five-year period after the study is finished. The information will be coded for handling and storage during the research process, and only the authors will have access to it. The information collected will be presented in aggregate form in publications and scientific conferences.*

4. Your ethics statement should only appear in the Methods section of your manuscript. If your ethics statement is written in any section besides the Methods, please move it to the Methods section and delete it from any other section. Please ensure that your ethics statement is included in your manuscript, as the ethics statement entered into the online submission form will not be published alongside your manuscript.

**ANSWER**: We have moved the ethical statements to the Methods section.

5. Please include captions for your Supporting Information files at the end of your manuscript, and update any in-text citations to match accordingly. Please see our Supporting Information guidelines for more information: <http://journals.plos.org/plosone/s/supporting-information>

**ANSWER**: Done.

6. Please review your reference list to ensure that it is complete and correct. If you have cited papers that have been retracted, please include the rationale for doing so in the manuscript text, or remove these references and replace them with relevant current references. Any changes to the reference list should be mentioned in the rebuttal letter that accompanies your revised manuscript. If you need to cite a retracted article, indicate the article’s retracted status in the References list and also include a citation and full reference for the retraction notice.

**ANSWER**: We have no references to replace or modify. We formatted the references following the PlosOne format and using EndNote 20 (Plos style).

**REVIEWER #1:**

Congratulations on your hard work. The word ‘per cent’ in methods and findings section of abstract must be corrected. I found a few writing errors in all text. Other than that, I found the article carefully prepared and well-done.

**ANSWER**: We appreciate your review. We have corrected that writing error in the abstract and we checked for typos in the rest of the text.

______________________________________________

I look forward to hearing from you regarding our submission in **PlosOne**. I will be glad to answer to any further questions and comments that you may have.

Sincerely,

Felipe González-Seguel, PT, MSc
